# A microbiota and dietary metabolite integrates DNA repair and cell death to regulate embryo viability and aneuploidy during aging

**DOI:** 10.1126/sciadv.ade8653

**Published:** 2023-02-24

**Authors:** Robert Sonowal, Alyson I. Swimm, Francesca Cingolani, Noyonika Parulekar, Tesia L. Cleverley, Anusmita Sahoo, Ayush Ranawade, Debalina Chaudhuri, Edward S. Mocarski, Heather Koehler, Karolina Nitsche, Sam Mesiano, Daniel Kalman

**Affiliations:** ^1^ Department of Pathology and Laboratory Medicine, Emory University School of Medicine, Atlanta, GA, USA.; ^2^ Immunology and Molecular Pathogenesis Graduate Program, Emory University, Atlanta, GA, USA.; ^3^ Emory Vaccine Center, Emory University, Atlanta, GA, USA.; ^4^ Yerkes National Primate Research Center, Emory University, Atlanta, GA, USA.; ^5^ Department of Microbiology and Immunology, Emory University School of Medicine, Atlanta, GA, USA.; ^6^ Department of Biology, Northeastern University, Boston, MA, USA.; ^7^ Mouse Transgenic and Gene Targeting Core, Emory University, Atlanta, GA, USA.; ^8^ Department of Reproductive Biology, Case Western Reserve University and Department of Obstetrics and Gynecology, University Hospitals of Cleveland, Cleveland, OH, USA.

## Abstract

During aging, environmental stressors and mutations along with reduced DNA repair cause germ cell aneuploidy and genome instability, which limits fertility and embryo development. Benevolent commensal microbiota and dietary plants secrete indoles, which improve healthspan and reproductive success, suggesting regulation of germ cell quality. We show that indoles prevent aneuploidy and promote DNA repair and embryo viability, which depends on age and genotoxic stress levels and affects embryo quality across generations. In young animals or with low doses of radiation, indoles promote DNA repair and embryo viability; however, in older animals or with high doses of radiation, indoles promote death of the embryo. These studies reveal a previously unknown quality control mechanism by which indole integrates DNA repair and cell death responses to preclude germ cell aneuploidy and ensure transgenerational genome integrity. Such regulation affects healthy aging, reproductive senescence, cancer, and the evolution of genetic diversity in invertebrates and vertebrates.

## Teaser

Plant- or microbiota-derived indoles repair DNA damage or induce cell death depending on stressor level to control embryo quality.

## INTRODUCTION

As animals age, germ cells exhibit increased aneuploidy and genomic instability, thereby reducing embryo viability and allowing the accumulation of mutations causing birth defects that ultimately limit fecundity and reproductive span (*1*, *2*). The germline DNA damage response (DDR) comprises cell cycle checkpoint regulators, DNA repair enzymes, and apoptotic proteins that ensure genome integrity (*3*). Upon sensing DNA damage, cell cycle checkpoints delay cell division and facilitate decisions to either repair the damage or initiate cell death to eliminate damaged embryos, a process ensuring faithful transmission of genetic information across generations (*4*, *5*). DDR pathways often become dysregulated with age, raising the possibility that reduced capacity for DNA damage surveillance, repair, or cell death may contribute to increased rates of aneuploidy and reduced fecundity evident in older individuals (*6*).

Although the DDR is well studied (*7*), much less is known about how checkpoints, DNA repair, and cell death mechanisms are integrated. Moreover, in animals where DDR becomes dysregulated with age, whether retuning or extending the function of this response can benefit fecundity is unknown. Recent data suggest that small molecules produced by commensal microbiotas or introduced as dietary supplements may promote extended periods of health, motility, and even fecundity, collectively termed healthspan (*8*, *9*). In line with this benefit, dysbiosis, the reduction in microbial diversity and loss of beneficial bacteria, has been associated with DNA damage, genomic instability, and cancer (*10*). However, whether or how dietary or microbiota factors that extend healthspan can affect germ cell quality remains unknown. To test this idea, we chose the nematode *Caenorhabditis elegans* as a biosensor and indoles, molecules derived from dietary sources or the microbiota that extend healthspan in vertebrates and invertebrates (*8*). *C. elegans* present an advantageous means to test these ideas, as they eat bacteria, and male frequencies together with embryo lethality serve as readily quantifiable readouts of aneuploidy on the X chromosome and autosomes, respectively (*11*, *12*). Animals cannot synthesize indoles and instead rely on dietary components, such as cruciferous vegetables, as well as the intestinal microbiota via tryptophanase (tnaA)–mediated catalysis of tryptophan (*8*). In mammals, indoles enhance the integrity of the epithelial barrier, thereby limiting dissemination of bacteria and bacterial antigens (*13*–*16*), reduce deleterious inflammation (*17*, *18*), and enhance motility in the aged (*8*). In *C. elegans*, indoles also extend the reproductive period, allowing aged animals to produce viable embryos for longer (*8*), suggesting that these molecules may affect germ cell quality. Here, we show that in aging germ cells from both vertebrates and invertebrates, indoles repair DNA damage or initiate cell death, depending on the level of damage, thereby maintaining ploidy and increasing genome integrity in the embryo. In so doing, indoles increase the proportion of viable embryos, which promotes fecundity across generations.

## RESULTS

### Indoles limit intergenerational embryo lethality and male frequency induced by environmental stressors in *C. elegans*

**Fig. 1.**
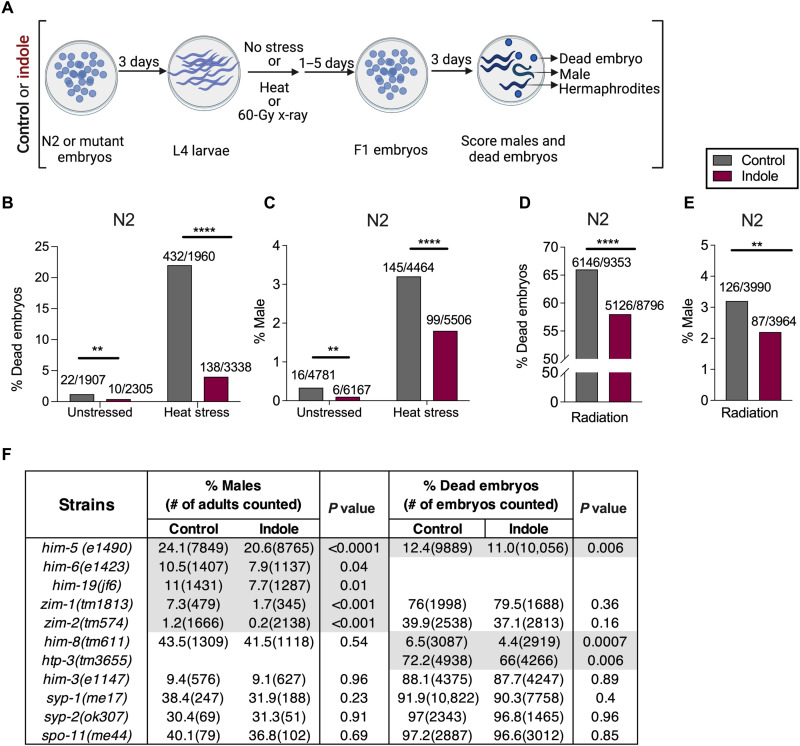
Indoles limit intergenerational embryo lethality and male frequency induced by heat stress, X-irradiation, and mutations in *C. elegans*. (**A**) Schematic for assessing male frequency and embryo lethality in the progeny of N2 or mutant worms grown either under control or under indole conditions. Indole or carrier was provided throughout the experiment. (**B** and **D**) Percentage of dead embryos produced by N2, subjected to either 30°C (B) or 60 Gy (D). (**C** and **E**) Frequency of males in the progeny of N2 exposed to either 30°C (C) or 60 Gy (E). Values on bars in (B) and (D) represent the number of dead embryos/total embryos counted, and bars in (C) and (E) represent the number of males/total live progeny counted. (**F**) Frequency of males and dead embryos produced by mutant worms. *P* values calculated by chi-square test. Combined results from at least two independent experiments are presented. **P < 0.01and ****P < 0.0001.

We evaluated the effects of indole on male frequencies and embryo lethality in the progeny of animals subjected to heat stress or X-irradiation. Wild-type (N2) worms were exposed to indole or carrier (MeOH) from the embryo stage onward (see Materials and Methods). Animals at the fourth larval (L4) stage were either left unstressed or subjected to transient heat stress and then allowed to deposit embryos (F1) at the permissive temperature. The sex and viability of their progeny were then enumerated. The indole or carrier treatment was continued for the duration of the experiment in both F0 and F1 generations (Fig. 1A). Heat stress alone increased the numbers of both males and dead embryos [compare gray bars in Fig. 1 (B and C)]. Under both normal growth conditions and heat stress, indole reduced the number of males and the number of dead embryos [compare gray and red bars in Fig. 1 (B and C)]. The reduction in male frequencies was too small in magnitude to permit resolution of changes in numbers of diakinetic chromosomes in the proximal gonad, an assay commonly used to assess aneuploidy in *him* strains (*11*, *12*). Similar to heat stress, X-irradiation [60 gray (Gy)] increased both the number of male progeny and the number of dead embryos [compare gray bars in Fig. 1 (B and C) with Fig. 1 (D and E)]. As with heat stress, exposure to indole reduced the number of dead embryos and the frequency of males [compare gray and red bars in Fig. 1 (D and E)]. Together, these data indicate that indoles reduce intergenerational aneuploidy associated with environmental stressors.

### Indoles limit intergenerational embryo lethality and male frequency due to mutations in *C. elegans*

Induction of males with stressors reads out X-chromosomal aneuploidy, but embryo lethality may result from other factors besides autosomal nondisjunction. To address this, we evaluated the effects of indole on male frequency and embryo lethality in "high incidence of males" (*him*) mutant strains, which produce males and dead embryos due to X chromosome and autosome nondisjunction, respectively (*11*). The *him* mutants used affect critical stages of chromosomal segregation and DNA repair during meiosis, including chromosomal pairing and attachment to the nuclear envelope (*him-8*, *zim-1*, and *zim-2*); homologous chromosome alignment and synapsis formation (*him-3*, *htp-3*, *syp-1*, and *syp-2*); homology recognition and double-stranded break (DSB) formation (*spo-11*, *him-19*, and *him-5*); crossover formation, recombination, and resolution of Holliday junction (*him-5* and *him-6*); and DSB repair (*him-5*) (*11*, *19*–*21*). Mutant strains were grown either in indole or in control but without exposure to stressors, and their progeny was enumerated. Indole reduced male frequencies in animals with mutations in *him-5*, *him-6*, *him-19*, *zim-1*, and *zim-2*, indicating suppression of nondisjunction on the X chromosome (gray highlight; Fig. 1F). Indole also decreased the number of dead embryos in animals with mutations in *htp-3*, *him-8*, and *him-5*, indicative of an effect of indole in limiting autosomal nondisjunction (gray highlight; Fig. 1F). Indole was without effect on male frequencies in *him-8* mutants, likely because indole did not affect X chromosome pairing in a mutant defective in that process. For similar reasons, indole was likely without effect on embryo lethality in *zim-1* or *zim-2* mutants, which govern autosomal pairing. In some mutants (e.g., *syp-1*, *syp-2*, *spo-11*, and *him-3*), indole did not alter male frequencies or embryo viability. However, the number of viable animals was so low as to preclude evaluation of enough animals to achieve significance. As a control, indole did not affect total brood size in N2 or in all mutants except *htp-3*(*tm3655*), *syp-1*(*me17*), and *syp-2*(*ok307*) (fig. S1, A and B). Together, data showing suppression of males or embryo lethality in several *him* mutants indicate that indole limits nondisjunction on both the X chromosome and autosomes and acts at multiple stages of meiosis.

### Indoles act via the DDR pathway to limit aneuploidy and embryo lethality in *C. elegans*

**Fig. 2.**
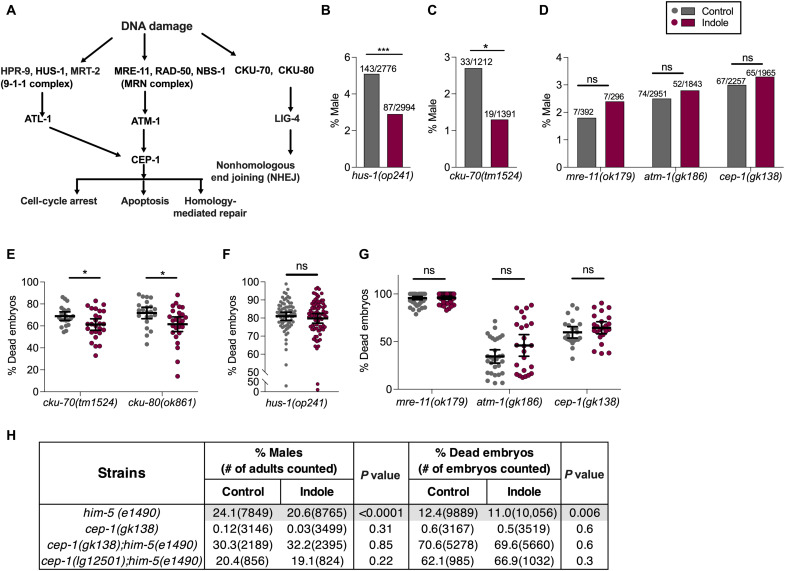
Indoles require the MRN pathway genes to limit male frequency and embryo lethality in *C. elegans*. (**A**) Schematic of DNA damage response (DDR) pathways in *C. elegans*. In *C. elegans* germline, DNA DSBs are detected by either the HPR-9-HUS-1-MRT-2 (9-1-1) sensor complex or by the MRN sensor complex, which activate the *C. elegans* p53 homolog CEP-1. An alternative DSB sensor induces nonhomologous end joining (NHEJ) in somatic cells, although this pathway is suppressed within the gonad (*64*). The MRN-p53/CEP-1 pathway regulates homology-mediated DSB repair during meiosis, and following genotoxic stress (*39*), p53/CEP-1 induces apoptosis via apoptosis-activating factor-1 (APAF-1) homolog and a caspase (*20*, *65*). (**B** to **G**) DDR mutant worms were grown in either control or indole, exposed to 60-Gy x-ray. Indole or carrier was provided throughout the experiments. (B to D) Frequencies of males in the progeny (values on bars represent total number of males/total live progeny counted). (E to G) Viability of embryos produced (*n* > 25 biological replicates/condition). (**H**) Male frequency, and embryo lethality in the progeny of worms harboring DDR mutations, and grown either under control or under indole conditions. Indole or carrier was provided throughout the experiments. *P* values in (B) to (D) and (H) calculated by chi-square test. Values in (E) to (G) represent mean values ±95% CI, and *P* values were calculated by Mann-Whitney test. Combined results from at least two independent experiments are presented. *P < 0.05 and ***P < 0.001. ns, not significant.

Both irradiation and indole-suppressible *him* mutants are associated with DNA strand breaks or repair during recombination (*20*, *22*, *23*), raising the possibility that indole regulates male numbers and embryo viability via DNA damage sensing pathways or the p53/*C. elegans *p53-like protein-1 (CEP-1) effector (Fig. 2A). To test this, strains containing inactivating mutations in components of the 9-1-1, nonhomologous end joining (NHEJ), MRE-11-RAD-50-NBS-1 (MRN) sensors, or CEP-1 effector were grown in the presence or absence of indole and subjected to X-irradiation, and the numbers of males and dead embryos in their progeny were enumerated as in Fig. 1A. As in Fig. 1A, indole or carrier treatment was continued for the duration of the experiment in the F0 and F1 generations. Indole suppressed male numbers in strains with mutations in *hus-1* (Fig. 2B) and *cku-70*, which encode components of the 9-1-1 and NHEJ pathways, respectively (Fig. 2C). However, no suppression was evident in strains containing mutations in *mre-11* or *atm-1*, which encode members of the MRN/Ataxia telangiectasia mutated-1 (ATM-1) pathway (Fig. 2D). Indole also reduced embryo lethality in NHEJ pathway mutants (Fig. 2E), but not in the 9-1-1 and MRN pathway mutants (Fig. 2, F and G). Together, these data indicate that in response to radiation damage, indole suppresses nondisjunction on the X chromosome via the MRN pathway and limits embryo lethality via both the MRN and 9-1-1 pathways.

Under normal conditions, *cep-1* mutant animals exhibit neither the *him* phenotype nor a diminution in numbers of viable embryos [Fig. 2H; (*20*)]. However, X-irradiation of *cep-1* mutants increased male numbers and embryo death to levels comparable to those of irradiated N2 animals (Fig. 2, D and G). Indole did not affect the number of males or embryo viability in *cep-1* mutants following X-irradiation, indicating that *cep-1* mediates indole effects on both processes (Fig. 2, D and G). To determine whether *cep-1* mediates the effect of indole in limiting aneuploidy in meiotic mutants, indole effects were evaluated in two independently derived *C. elegans* strains harboring loss-of-function mutations in both *cep-1* and *him-5*. These strains exhibited a genetic interaction between *cep-1* and *him-5* such that double mutant animals displayed a significantly higher frequency of males and embryo lethality, compared with either single mutant strain or N2 [Fig. 2H; (*20*)]. Whereas indole suppressed male percentages and embryo lethality in *him-5*(*e1490*) animals (Figs. 1F and 2H), indole had no effect on either *cep-1;him-5* double mutant strain tested (Fig. 2H), indicating that indole-mediated suppression of *him-5* mutants requires p53/CEP-1. These data suggest that indole acts via Meiotic Recombination-11 (MRE-11), ATM-1, and p53/CEP-1 to limit aneuploidy in response to stressors or mutation.

Besides DNA repair, the DDR pathway also induces cell cycle arrest and apoptosis following damage [Fig. 2A; (*23*)]. Indole did not affect the number of mitotic nuclei in the distal tip region of the gonad following exposure to 60-Gy X-irradiation, suggesting the absence of its influence on cell cycle arrest (fig. S2A). Likewise, no increase in numbers of apoptotic cells, as measured by acridine orange staining, was evident in the pachytene region of the ovary following radiation exposure (60 Gy; fig. S2B). However, indole-induced apoptosis was not easily resolved by enumerating apoptotic cells within the gonad, where only a few apoptotic cells per animal were ever evident. To determine a role for apoptosis, strains harboring loss-of-function mutations in the core apoptotic pathway genes *ced-3*, which encodes a caspase, and *ced-4*, which encodes an APAF-1 homolog, were grown in indole and then subjected to 60-Gy X-irradiation. In both strains, indole-mediated suppression of male numbers and embryo lethality was similar to that observed in N2 animals [compare Fig. 1 (D and E) and fig. S2 (C to E)]. These data indicate that indole limits aneuploidy via MRE-11, ATM-1, and p53/CEP-1 to induce repair of DSBs, and at the 60-Gy dose level, no role for indole in regulating apoptosis or checkpoint-mediated cell cycle arrest was evident.

### Indoles limit age-dependent intergenerational increases in males and reduces embryo lethality in *C. elegans*

**Fig. 3.**
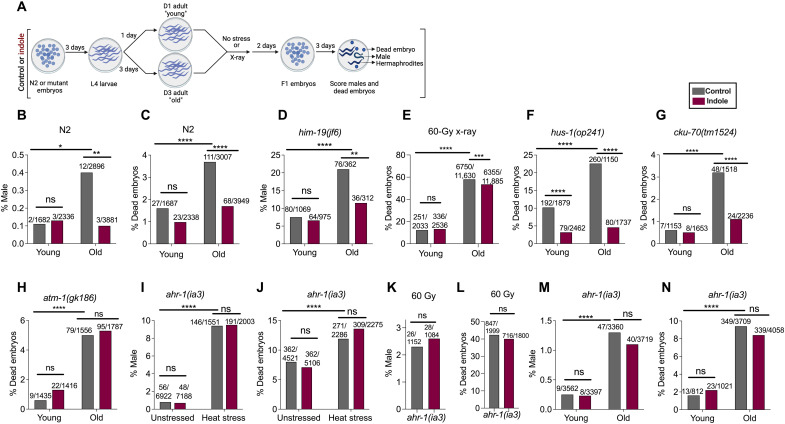
Indoles limit incidence of male and embryo lethality associated with stressor or aging in *C. elegans* via MRN and Ahr-1 pathways. (**A**) Schematic for assessing male frequency and embryo lethality induced with aging or stressors in *C. elegans*. Worms were grown either under control or under indole conditions for the duration of all experiments. Frequencies of male or dead embryos in the progeny of young [day 1 (D1) to day 3 (D3) of adulthood] and old (D3 to D5 of adulthood) N2 (**B** and **C**) or *him-19* mutant animals (**D**). (**E**) Percentage of dead embryos in the progeny from young and old N2 worms, subjected to 60 Gy, and allowed to lay embryos for 48 hours. (**F** to **H**) Frequencies of dead embryos in the progeny of young and old DDR mutant worms. Frequency of males (**I** and **K**) and dead embryos (**J** and **L**) in the progeny of *ahr-1*(*ia3*) worms, subjected to either 30°C heat stress (I and J) or 60 Gy (K and L). Frequencies of males (**M**) and dead embryos (**N**) in the progeny of young and old *ahr-1*(*ia3*) worms. Values on the top of the bars represent total number of males/total live progeny counted in (B), (D), (I), (K), and (M), and number of dead embryos/total embryos counted in (C), (E) to (H), (J), (L), and (N). Combined results from at least two independent experiments are presented. *P* values calculated by chi-square test. *P < 0.05, **P < 0.01, ***P < 0.001, and ****P < 0.0001.

The incidence of males and embryo lethality in the progeny increase as animals age, an effect attributed to increased oocyte aneuploidy (*24*, *25*). To evaluate the effects of indole on aneuploidy during normal aging, the incidence of males and dead embryos produced by young [day 1 (D1) to D3] and old (D3 to D5) N2 animals was compared (Fig. 3A). Whereas indole had no effect in young animals, it reduced male frequencies and embryo lethality in old animals to levels similar to that seen in young animals (Fig. 3, B and C). *him-19* mutants have been previously found to exhibit a twofold increase in the frequency of males as they age (*21*). Whereas indole had no detectable effect on male frequencies in young *him-19* mutants, it reduced the frequency of males in old D3 to D5 animals to a level seen in young animals (Fig. 3D). Effects of indole on embryo lethality were also assessed following X-irradiation of young and old N2 animals. Animals were irradiated at either D1 or D3, and the viability of their embryos was evaluated over the next 2 days. Embryo lethality associated with radiation increased significantly from D1 to D3 (Fig. 3E). Whereas indole did not affect the viability of embryos from D1 animals, it significantly reduced embryo lethality in D3 animals (Fig. 3E). Although radiation increases the number of males, an effect of indole on male frequencies with aging was not assessed because males occurred at too low a rate in the 48-hour period when embryo viability was evaluated.

To decipher the genetic pathway mediating the effects of indole during aging, the embryo lethality of mutants in the 9-1-1 pathway [*hus-1*(*op241*)], NHEJ pathway [*cku-70*(*tm1524*)], and MRN pathway [*atm-1*(*gk186*)] grown with or without indole was compared at D1 and D3. Unlike the *hus-1*(*op241*) and *cku-70*(*tm1524*) mutants (Fig. 3, F and G), indole had no effect on age-dependent increases in embryo lethality in the MRN pathway mutant *atm-1*(*gk186*) (Fig. 3H). These data indicate that old animals are more susceptible to damage associated with environmental stressors such as radiation, various mutations (e.g., *him-19*) have a more pronounced impact on nondisjunction in aged animals compared to younger ones, and indole reduces male frequencies and increases embryo viability in old animals. These data also indicate that indole acts via the MRN pathway to suppress age-dependent increases in embryo lethality.

### Indoles act via the AhR to regulate nondisjunction in *C. elegans*

Previous studies showed that aryl hydrocarbon receptor (AhR) mediates the effects of indole on healthy aging (*8*, *15*), raising the possibility that indole also acts via AhR to regulate aneuploidy and embryo lethality. *ahr-1*(*ia3*) mutant worms display a significantly higher frequency of male progeny compared to N2 animals in both unstressed [0.8% in *ahr-1*(*ia3*) versus 0.3% in N2] and heat-stressed [9.4% in *ahr-1*(*ia3*) versus 3.2% in N2] conditions (compare Figs. 1C and 3I). Whereas indole reduced male frequencies in N2 animals, it had no effect in *ahr-1*(*ia3*) animals (compare Figs. 1C and 3I). The *ahr-1*(*ia3*) animals exhibited higher embryo lethality without stress compared to N2, an effect that was exacerbated by heat stress (Figs. 1B and 3J). In contrast to N2, indole did not abrogate embryo lethality with or without heat stress in *ahr-1*(*ia3*) animals (Figs. 1B and 3J). Indole also failed to suppress radiation-induced increases in numbers of males and embryo lethality in *ahr-1*(*ia3*) mutants (Fig. 3, K and L). Indole also did not affect age-dependent increases in the percentage of males and embryo lethality in *ahr-1*(*ia3*) mutants (Fig. 3, M and N). The absence of an indole effect in these various conditions was recapitulated in a second loss-of-function *ahr-1* mutant, *ahr-1*(*ju145*) (fig. S3, A to C). Together, these data indicate that indole acts via *ahr-1* to limit aneuploidy and promote embryo viability in response to both stressors and aging.

### ICA limits X-irradiation–induced DNA damage and promotes DNA repair in fibroblasts and splenocytes

**Fig. 4.**
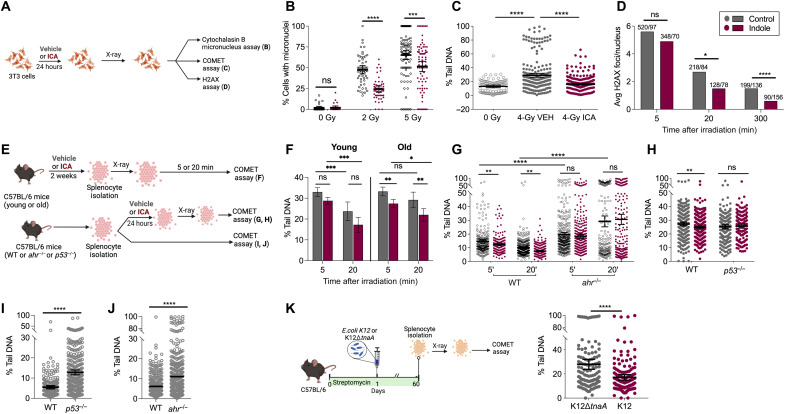
Indoles limit x-ray–induced DNA damage and promote DNA repair in fibroblasts and splenocytes. (**A**) Schematic of DDR assays. 3T3 fibroblast cells were treated with vehicle or ICA and assayed for the frequency of micronuclei with different radiation doses (**B**), percent comet tail DNA (*n* > 102 comets per condition; **C**), and H2AX foci after 4-Gy x-ray (**D**). Values above the bars represent number of foci observed/total nuclei counted. (**E**) Schematic of DDR assays in splenocytes from C57BL/6 mice. (**F** to **K**) The percentage (%) of tail DNA in comets. (F) Four-gray irradiated splenocytes isolated from ICA/vehicle-treated young (3-month) and old (18-month) C57BL/6 mice (*n* > 102 comets per condition). (G) Four-gray irradiated splenocytes from *ahr*^−/−^ mice (*n* > 200 comets per condition; comet assay performed 5′ and 20′ after radiation). (H) Four-gray irradiated splenocytes from *p53*^−/−^ mice (*n* > 170 comets per condition). (I) Splenocytes from *p53*^−/−^ mice without stress (*n* > 230 comets per condition). (J) Splenocytes from *ahr*^−/−^ (*n* > 360 comets per condition) and age-matched C57BL/6 mice without stress. (K) Four-gray irradiated splenocytes from C57BL/6 mice colonized with either K12 or K12∆*tnaA* (*n* > 200 comets per condition). Values represent combined results from at least two independent experiments. (B), (C), and (F) to (K) represent mean values ±95% CI. *P* values calculated with Mann-Whitney test (B, C, and G to K), Kruskal-Wallis (F) or chi-square test (D). *P < 0.05, **P < 0.01, ***P < 0.001, and ****P < 0.0001.

We next investigated whether the effects of indole on DDR and aneuploidy is conserved in mammalian somatic and germline cells. Previous experiments in mammals showed that the indole derivative indole-3-aldehyde (ICA) has a higher activity than indole, and both promotes healthspan and provides protection against intestinal damage caused by radiation, by pathogen exposure, or by allo-immune responses (*8*, *13*, *18*). Thus, ICA rather than indole was used in mammalian experiments. 3T3 fibroblasts were exposed to X-irradiation in the presence or absence of ICA, and the cytochalaisin B-micronucleus assay was used to quantify cells with unrepaired DNA damage, missegregated chromosomes, or chromosomal breakage [Fig. 4A; (*26*)]. ICA reduced the percentage of cells with one or more micronucleus at all X-irradiation doses tested, despite increased numbers of micronuclei at higher radiation doses (Fig. 4B).

The micronucleus assay cannot distinguish the effects of X-irradiation on spindles versus chromosomes, both of which could affect chromosome segregation. To directly assess the effects of ICA on single- or double-stranded DNA breaks, alkaline comet assays were performed on X-irradiated 3T3 cells to assess the electrophoretic mobility of nuclear DNA (Fig. 4). In a comet assay, cells are embedded in agar and electrophoresed, stained with 4′,6-diamidino-2-phenylindole (DAPI), and imaged. Imaged cells resemble a “comet” with a distinct head and tail. The head is composed of intact DNA, while the tail consists of damaged DNA with single-stranded breaks or DSBs or broken pieces of DNA. The extent of DNA liberated from the head into the tail of the comet is directly proportional to the amount of DNA damage.

Treatment with ICA reduced percent tail DNA, the tail moment, and the olive moment, all direct measures of the extent of DNA damage (Fig. 4C and fig. S4, A and B). To confirm that ICA regulated DNA repair, 3T3 cells were X-irradiated and, at various times, thereafter, stained with phospho-γ-H2A histone family member X (H2AX) monoclonal antibody, which recognizes DSBs. Compared to vehicle-treated cells, ICA had no effect on the number of γ-H2AX foci detected per nucleus at the 5-min time point (Fig. 4D). However, by 20 min, ICA decreased the number of foci compared to vehicle treatment, an effect further enhanced at 5 hours after irradiation (Fig. 4D). Histograms of the frequency of nuclear γ-H2AX foci per nucleus are shown in fig. S4 (E and F). Together, these data indicate that ICA promotes repair of DNA DSBs in mitotic mammalian cells.

We next evaluated the effects of ICA on DDRs in somatic cells during aging in vivo. To do this, 3- or 18-month-old C57Bl/6 mice were treated with either vehicle or ICA for 2 weeks, and their splenocytes were isolated, X-irradiated, and subjected to alkaline comet assays 5 or 20 min later (Fig. 4E, top). A reduction in comet tail DNA was evident when comparing the 5- and 20-min time points in young animals treated with vehicle, indicative of active repair of DNA damage, and ICA did not provide additional benefit (Fig. 4F, left). By contrast, limited repair was evident between the 5- and 20-min time points in old animals treated with vehicle, but ICA reduced comet tail DNA at both time points (Fig. 4F, right). These data indicate that splenocytes from young animals retain high DNA damage repair activity, which decreases with age, and ICA restored repair activity in old animals to levels seen in young ones. These data also rule out the possibility that indole effects on repair are restricted to immortalized cells.

### ICA acts via AhR and p53 to limit X-irradiation–induced DNA damage in mammals

To determine whether effects of ICA on DDR in mammals depend on AhR and p53, splenocytes were isolated from 20-month-old *ahr*^−/−^ mice and their age-matched C57Bl/6 wild-type counterparts or 2-month-old *p53*^−/−^ mice and their age-matched C57Bl/6 counterparts (Fig. 4E, bottom). Splenocytes were treated ex vivo with ICA, X-irradiated, and subjected to comet assays. Whereas ICA reduced comet size in splenocytes from the C57Bl/6 animals, it had no effect on splenocytes derived from *ahr*^−/−^ or *p53*^−/−^ animals (Fig. 4, G and H). Moreover, tail percentages were higher in splenocytes from *ahr*^−/−^ and *p53*^−/−^ animals even without radiation compared to those from the C57Bl/6 mice (Fig. 4, I and J), indicating a higher level of baseline damage, lower repair potential, or both. Together, these data indicate that ICA acts via AhR and p53 to facilitate repair of damaged DNA and that these genes mediate protective effects of indoles in vivo.

### Commensal *E. coli* protect mammalian splenocytes from X-irradiation–induced DNA damage

We next investigated whether gnotobiotic colonization of the mammalian gut with a commensal bacterial strain producing indoles exerted a DDR akin to that of enteral administration of ICA (see Fig. 4K for protocol). To do this, C57Bl/6 mice were treated with streptomycin to reduce the microbial diversity and numbers within the intestinal tract (*8*) and recolonized with either Strp^R^ *E. coli* K12, which produces indole and indole derivatives, or Strp^R^ *E. coli* K12∆*tnaA*, which does not (*8*). The mice maintained the strains for up to 3 months (*8*). After 2 months, splenocytes were isolated, X-irradiated, and subjected to comet assays 20 min later. Splenocytes derived from K12-colonized animals exhibited lower percentage tail DNA and reduced tail and olive moments compared to those in splenocytes from K12∆*tnaA*-colonized mice (Fig. 4K and fig. S4, C and D). These data indicate that indoles derived from a commensal *E. coli* strain limit radiation-induced DNA damage.

### ICA limits DNA damage in young mammalian oocytes

**Fig. 5.**
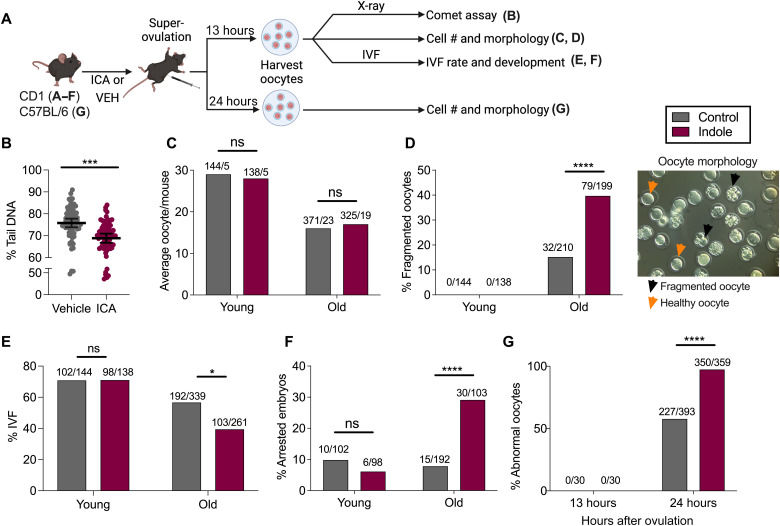
Indoles regulate DNA repair and survival of mouse oocytes. (**A**) Schematic of mouse oocyte quality assessment. (**B**) Percentage of tail DNA in the comets of X-irradiated CD1 oocytes from mice treated with ICA or vehicle (*n* > 70 comets per condition). (**C** to **F**) Quality assessment of the oocytes from young (3 months old) or old (> 6 months) CD1 mice treated with ICA or vehicle. (C) Oocyte number per mouse. Values above the bars represent the number of oocytes/total number mice. (D) Frequency of fragmented oocytes. Values above the bars represent the number of fragmented oocytes/total oocytes. The right panel in (D) represents morphologies of normal versus fragmented (abnormal) oocytes (magnification, ×40). (E) The IVF success rate. Values above the bars represent the number of fertilized oocytes/total number of oocytes. (F) Frequency of developmental arrest observed in embryos after IVF. Values above the bars represent the number of arrested embryos/total embryos scored. (**G**) Frequency of abnormal oocytes from young (3 months old) C57BL/6 mice ICA/vehicle treated for 2 days and subjected to post-ovulatory aging. Values above the bars represent number of abnormal oocytes/total number of oocytes. Data represent combined results from at least two independent experiments. In (B), data represent mean values ±95% CI. *P* values were calculated with Mann-Whitney test (B) or chi-square test (C to G). *P < 0.05, ***P < 0.001, and ****P < 0.0001.

Observations in splenocytes suggested that ICA facilitates DNA repair in mitotically dividing mammalian somatic cells. To determine whether ICA likewise limited DNA damage in mammalian germline cells, especially during aging, DDRs were next assessed in mammalian oocytes (Fig. 5A). To collect oocytes, 3-month-old CD1 mice were administered either ICA or vehicle for up to 2 weeks, treated with pregnant mare serum gonadotropin (PMSG) followed by human chorionic gonadotropin (hCG) to induce superovulation. Oocytes were then collected from the ampulla, X-irradiated (4-Gy), allowed to recover for 5 min, and then subjected to alkaline comet assays. As shown in Fig. 5B, oocytes derived from mice treated with ICA exhibited reduced percentage of tail DNA compared to vehicle controls, indicating that, as with mitotic cells, ICA limited the sensitivity of oocytes to DNA damage from X-irradiation.

### ICA induces fragmentation and reduces viability of oocytes from aged mammals

To determine whether indole regulates mammalian oocyte quality with aging, we evaluated the morphology of the oocytes following ovulation and monitored subsequent development following in vitro fertilization (IVF) (Fig. 5A). First, we exposed 3-month-old (young) CD1 mice to ICA or carrier for up to 2 weeks, harvested oocytes from the ampulla 13 hours after superovulation, and then assessed their morphology and the capacity to form blastocysts following IVF with sperm derived from 3-month-old males. Young animals yielded large numbers of intact oocytes (~28 per animal) 13 hours after induction of superovulation with hCG (Fig. 5C). Oocytes with abnormal morphology such as fragmentation were not observed (Fig. 5D), and, upon IVF, 70% of the oocytes were successfully fertilized, as assessed by the appearance of two polar bodies and two distinct pronuclei 8 to 10 hours later (Fig. 5E). Most of the fertilized oocytes developed into blastocyst after 96 hours with only 10% of the embryos exhibiting developmental arrest (Fig. 5F). The oocytes derived from mice treated with ICA exhibited similar scores in all these assays (Fig. 5, C to F), indicating the absence of an effect in young mice.

We next assessed the effects of indoles on oocytes derived from 8-month-old (old) CD1 female mice. Notably, oocytes from this strain exhibit loss of quality by 6 to 9 months of age. Old CD1 females were administered ICA or vehicle for up to 2 weeks. Upon superovulation, the number of oocytes recovered from older animals was significantly reduced compared to young animals. As with young animals, pretreatment of old mice with ICA was without effect on oocyte yield (Fig. 5C). Thus, ICA did not affect the age-associated decline in the number of oocytes induced to ovulate with hormone treatment. However, oocytes derived from old animals administered ICA exhibited significantly higher rates of fragmentation, compared to rates from vehicle-treated animals (Fig. 5D). Moreover, upon IVF, fertilization rates were markedly reduced, and developmental arrest was evident in a higher fraction of fertilized oocytes derived from ICA-treated animals as compared to rates from vehicle-treated animals (Fig. 5, E and F). Although numbers were small, no differences were evident in the capacity of blastocysts derived from ICA- or vehicle-treated oocytes to implant or generate viable pups. Therefore, somewhat unexpectedly, these data indicated that ICA reduced the viability of oocytes derived from aged animals and limited the developmental potential of embryos.

To determine whether the effects of ICA were specific to CD1 mice or associated with aging per se, we next assessed its effects on oocytes derived from young C57Bl/6 females, previously treated with ICA or vehicle. Oocytes were allowed to mature in the ampulla for 24 hours instead of 13 hours after superovulation. This protocol, called “postovulatory aging,” induces fragmentation in oocytes and recapitulates the aging of oocytes in the absence of sperm or fertilization [Fig. 5A; (*27*)]. While ICA did not affect young oocytes harvested at 13 hours (Fig. 5C), prior exposure to ICA of oocytes harvested at 24 hours resulted in an increased frequency of oocytes displaying fragmentation or abnormal morphology compared to vehicle controls (Fig. 5G). Together, these data indicate that whereas ICA was without effect on fragmentation or postfertilization development of oocytes harvested from young animals, it increased oocyte fragmentation and reduced viability of oocytes subjected to the postovulatory aging protocol.

### Exposure to indoles during late adulthood reduces viability of embryos from aged *C. elegans*

**Fig. 6.**
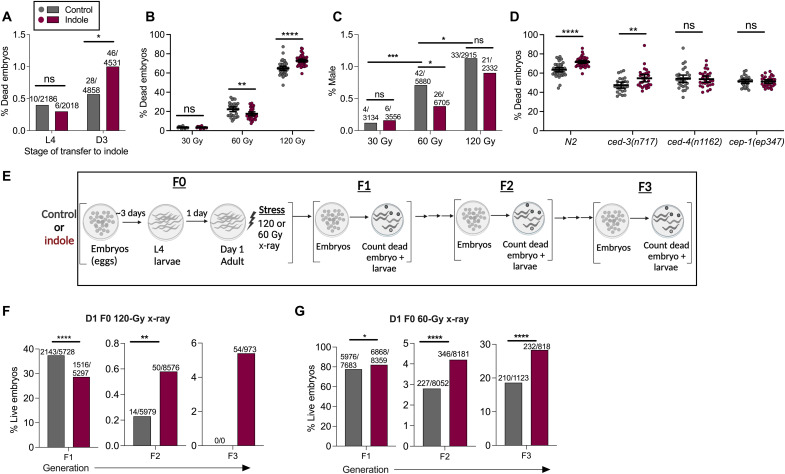
Indoles regulate DNA repair and cell survival depending on age and level of damage to promote transgenerational survival in *C. elegans*. (**A**) Frequency of dead embryos from N2 grown under control conditions and transferred to indole condition either at the L4 or D3 stage (values represent “number of dead embryos/total number of embryos”). (**B**) Frequency of dead embryos. (**C**) Male progeny from control/vehicle grown D1 adult N2, subjected to 30-, 60-, and 120-Gy x-ray [values in (C) represent “number of males/total number of adults scored”]. (**D**) Frequency of dead embryos from D1 N2 or *cep-1*, *ced-3*, and *ced-4* mutants subjected to 120-Gy x-ray. (**E**) Schematic of transgenerational embryo survival assays. (**F** and **G**) Frequency of live embryos obtained until F3 generation when F0 D1 N2 adults grown either in control or in indole were exposed to either 120 Gy (F) or 60 Gy (G) x-ray (values represent “number of live embryos/total number of embryos scored”). All values represent combined results from at least two independent experiments. Data in (B) and (D) represent mean values ±95% CI. *P* values were calculated with Mann-Whitney test (B and D) or chi-square test (A, C, F, and G). *P < 0.05, **P < 0.01, ***P < 0.001, and ****P < 0.0001

The observation that ICA limited DNA damage in young murine oocytes but promoted fragmentation in aged oocytes or in young oocytes subjected to the postovulatory aging protocol suggested that, during aging, DDR declines or damage levels increase, or both, and that indole may have different effects depending on the level of damage. To determine whether such effects were recapitulated in *C. elegans*, we first assessed whether indole exposure only during late adulthood resulted in reduced survival of embryos. To do this, worms were exposed to indole beginning at either L4 (young) or D3 of adulthood (aged), and the number of viable embryos was enumerated 48 hours later. Whereas indole had no effect on embryos from animals exposed to indole at L4, viability was strongly decreased in embryos from animals exposed at D3 (Fig. 6A). These data suggest that, in both worms and mammals, exposing aged animals to indole reduces the viability of oocytes or embryos.

### Indoles differentially regulate aneuploidy and embryo viability in *C. elegans* depending on the dose of X-irradiation

Next, to test whether indole limits DNA damage or alternatively promotes cell death depending on the level of damage, we compared the effects of 30-, 60-, or 120-Gy X-irradiation on male frequencies and embryo viability in *C. elegans*. Increasing doses of radiation from 30 to 120 Gy without indole increased both frequency of males and embryo lethality, indicating increased aneuploidy (Fig. 6, B and C). Indole was without effect at 30 Gy but decreased both male frequency and embryo lethality following exposure to 60 Gy (Fig. 6, B and C). However, at 120 Gy, indole increased embryo lethality but did not reduce frequencies of males (Fig. 6, B and C). To decipher the pathway involved in the embryo lethality observed with indole at 120 Gy, mutant strains of the DNA damage checkpoint, p53/*cep-1*, and its downstream cell death effectors *ced-3* and *ced-4* were considered. Effects of indole on embryo lethality at 120 Gy were abrogated in animals with mutations in *cep-1* and *ced-4*, but not in *ced-3* (Fig. 6D), suggesting that the observed effects depended on p53/CEP-1 effector and the apoptosis activator cell death abnormality-4 (CED-4), but not on the CED-3 protease. Together, these data indicate that indole acts via the *p53/cep-1* and *ced-4* pathway to differentially regulate embryo viability and aneuploidy depending on the level of stressor-induced damage.

### Indoles increase transgenerational viability in *C. elegans*

We next assessed whether the differential regulation of embryo fate by indole at different levels of damage affects the overall fitness of the population across generations. To assess transgenerational viability, we irradiated control or indole-treated F0 animals with either 60 or 120 Gy at D1 of adulthood and assessed the viability of F1 embryos and the reproductive potential of the F1 offspring and offspring of subsequent generations (Fig. 6E). For these experiments, indole exposure or lack thereof was maintained in subsequent generations. At 120 Gy, only 37% of F1 embryos of control animals were viable, and indole further reduced viability to 28% (Fig. 6F). Of the viable F1 offspring, those derived from control F0 animals exhibited 47% sterility, compared to 26% from indole-treated F0 animals. F2 embryos produced by nonsterile F1 animals showed significantly reduced viability compared to F1 embryos, but those derived from indole F1 animals were 3.2-fold more viable than those derived from control F1 animals (Fig. 6F). Few control F2 embryos hatched, and these were sterile and generated no viable offspring. By contrast, F3 embryos from indole-treated F2 animals showed 4% viability. Thus, although F1 embryos derived from indole-treated F0 parents exhibited more lethality at 120 Gy compared to control animals, F2 and F3 offspring from these animals were more viable than those from control F0 animals (Fig. 6F). At 60 Gy, indole exposure produced a different effect in F1 embryos compared to that seen at 120 Gy (Fig. 6F), increasing the viability to 87% compared to 84% in controls (Fig. 6G). No sterility was evident in the F1 animals whether or not they were exposed to indole, but indole F2 or F3 embryos exhibited greater survival compared to control F2 or F3 embryos (Fig. 6G). Thus, lethality of F1 embryos decreased or increased with indole depending on the level of X-irradiation. Nevertheless, at both irradiation levels, indole increased the viability of embryos in subsequent generations. Together, these data suggest that indole differentially regulates embryo viability depending on the level of damage but nevertheless increases population fitness in subsequent generations.

## DISCUSSION

Data presented here indicate that indole, a small molecule produced by commensal microbiota or by dietary plants, regulates cellular repair and cell death mechanisms in worms and mammals to limit aneuploidy and increase genome integrity in the germline, so as to promote high-fidelity transmission of genetic information across generations. These data highlight the importance of an ancient interkingdom signaling molecule in promoting genetic homeostasis or, in its absence, driving genetic variation.

### Indole regulates DNA repair via AhR, the DDR pathway, and p53

Data presented here indicate that the indoles regulate the sensitivity of the DDR in both meiosis and mitosis to promote genome integrity, limit aneuploidy, and thereby ensure genome quality of daughter cells. In *C. elegans*, the DDR factors MRE-11, ATM-1, and p53/CEP-1 mediate the protective response of indole following radiation, mutation, or aging (Figs. 2 and 3), in line with previous reports showing that homology-mediated DNA repair via MRN is the primary detection and repair mechanism in the germline (*20*). Our data with the *hus-1* mutant suggest an additional role for the 9-1-1 pathway in mediating indole effects (Fig. 2). Vermezovic *et al.* (*28*) showed that the 9-1-1 pathway and NHEJ mediate DNA repair responses in somatic cells and tissues, particularly in adults and developing embryos, raising the possibility that the effects of indoles act on cells outside the germline.

Protective effects of indole against radiation or aging require AhR, a phenotype conserved across diverse phyla (Figs. 3 and 4). In accordance with our data, previous reports suggest that in immortalized cells, ionizing radiation acts via H2AX, DNA-protein kinase (PK), and ATM to promote DNA repair (*29*), an effect that also depends on AhR. These data are in line with previous reports from us and others showing that ICA acts via AhR to limit apoptosis and promote radioprotection (*18*, *30*). Besides ICA and indole, other indole derivatives likewise promote radioprotection both in vitro and in vivo. Notably, the extent of radioprotection and cell death with indoles varies across cell lines (*31*–*33*), raising the possibility that differential expression of antiapoptotic factors determines the threshold of radioprotection. Our data suggest that indole acts via cellular mechanisms involving AhR interfacing with chromosomal DDR and repair machinery. However, a more precise mechanism by which indole detects DNA damage and induces repair awaits biochemical characterization of indole effects on these processes.

### Tuning the indole response to regulate DNA repair versus cell death in worms and mammals

The observation that indole had opposite effects on embryo lethality at 60 Gy versus 120 Gy (Fig. 6, F and G) raised the possibility that indole senses both the level of DNA damage and the cellular repair capacity and thus acts as a rheostat to either initiate repair or induce cell death. Both mechanisms depend on p53, but the cell death pathway additionally depends on CED-4, which encodes the worm homolog of APAF-1, an activator of apoptosis (*34*), but does not depend on the CED-3 caspase (Fig. 6B). In this regard, CED-4 has been reported to activate a DNA damage checkpoint without apoptosis and to induce cell death in a manner independent of CED-3, possibly via other *C. elegans* caspases and signaling factors (*35*, *36*).

**Fig. 7.**
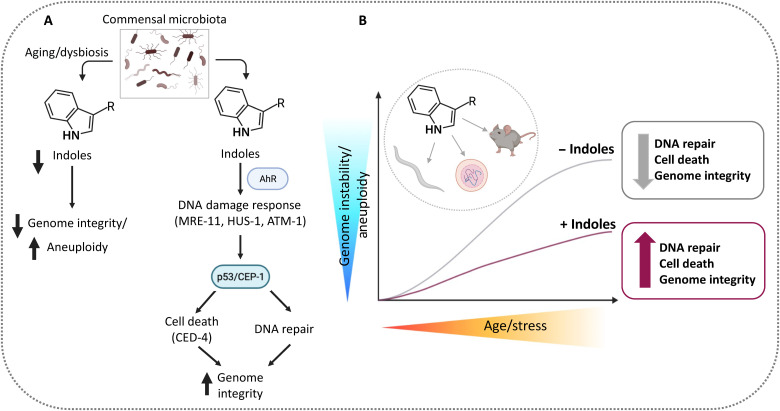
Model of indole quality control regulation. (**A**) Indole and the indole derivative ICA are derived from the microbiota or from dietary sources (e.g., cruciferous vegetables) and act via AhR, MRN-1, and p53/CEP-1 to detect DNA damage and activate the DSB repair machinery when damage is reparable, and cell death via the apoptosis activating factor CED4 when it is not, ensuring genome integrity and homeostasis. Alternatively, dysbiosis, dietary changes, or aging results in decreased levels of indole and thereby increased aneuploidy and genome variability. (**B**) Indoles act in a “quality control” capacity via MRN and p53 to affect genome integrity. In youth or low stressor levels, repair capacity is high, and indole has little effect. With age or increasing stressor levels, indole augments repair together with cell death, so that surviving cells have higher genomic integrity but more limited diversity compared to cells without indole. Thus, cell death removes heavily damaged cells, limiting their impact on diversity and fecundity. Increasing either repair or cell death increases the percentage of F1 animals with intact genomes, providing a survival advantage in successive generations. Limiting indole, which occurs with dysbiosis, dietary changes, or aging, relaxes the p53 checkpoint, resulting in decreased repair and cell death, increased aneuploidy, and, in worms, more males. Males outcross via sexual reproduction, increasing genomic diversity and allowing rapid adaptation to changing environments. Worm and mammalian oocytes have different set points for death and repair, with worms favoring survival and repair over death and mammals favoring the opposite. We speculate that species differences arise from selection for intact genomes when sequentially generating small numbers of highly complex live young, which is metabolically expensive, versus generating large numbers of embryos.

An extensive array of factors and mechanisms exist to stimulate cell death (e.g., caspases, APAF, etc.) or limit it (e.g., cellular inhibitor of apoptosis protein (cIAP), B cell lymphoma-2 (BCL-2), etc.) and to integrate extrinsic or intrinsic signals (*37*). Pathways favoring survival over death have been exploited by viruses to enable their unfettered replication without destruction of the host cell, which has likely facilitated the evolution of complex positive and negative signaling factors controlling cell survival (*38*). Data presented here suggest that molecules from microbiota and plants may likewise exert regulatory control over the DNA repair pathways to induce cell death on the one hand or repair and survival on the other, depending on the level of DNA damage and the cellular repair capacity. This regulation may be particularly important in allowing adaptation to change in the DNA repair capacity, which may decline with age or, alternatively, damage, which may increase with age (Fig. 7). The indole-mediated increase or decrease in embryo lethality improved viability in subsequent generations, highlighting the utility of the indole rheostat in promoting overall fecundity (Fig. 6). We propose that indole, together with the DNA damage sensing and response pathways, and apoptotic pathways act as a quality control regulator to ensure genome integrity. Indole may act via traditional DNA damage meiotic or mitotic checkpoints, a possibility suggested by our data showing that indole effects depend on MRN and CEP-1/P53 (Figs 2, 4, and 6). Indole appears to act in a manner that depends on the damage level to limit aneuploidy or promote cell death (Fig. 6). However, indole did not affect other cell cycle checkpoint phenotypes in the gonad such as cell cycle arrest of distal tip cells or cell death (*39*) at 60 Gy (fig. S2A), raising the possibility that indole regulates extant checkpoints in previously unidentified ways.

Meiosis in worms and mammals has remained generally conserved over 500 million years of evolution, but important species-specific differences exist, which appear to affect the tuning of indole regulation. Mammals exhibit far higher proportions of dead oocytes compared to worms, in part due to increased organismal complexity and the capacity to sequentially bear only a limited number of live offspring. In worms, half of the ~600 oocytes produced by an adult hermaphrodite survive. Remarkably, in women of reproductive age (~15 to ~55 years), ~19,999 of the 20,000 oocytes forming in the human ovary during a menstrual cycle undergo apoptosis each month until the supply of oocyte is exhausted at menopause. Overall, only 500 to 1000 oocytes are ovulated out of an initial cohort of ~1 million primordial oocytes, making the differential in usable oocyte rates between worms and humans 50% versus 0.1%, respectively. Rates in mice are more akin to humans, although the reproductive cycle is 4 days instead of a month, and females ovulate ~10 times the number of oocytes per cycle and bear ~10 times the number of offspring per pregnancy compared to humans.

While the incidence of cell death in mammalian oocytes is higher in mammals than in worms, in both types of animals, the probability of ovulating a viable oocyte diminishes with maternal age. This decline in oocyte viability coincides with an increased risk of aneuploidy, including Down’s syndrome (Trisomy 21) and Klinefelter syndrome (47, XXY), which increases significantly in incidence with parental age (*40*, *41*). In addition, offspring of aged female mice exhibit increased risk of metabolic problems (*42*). The decline in oocyte viability has been attributed to a decrease in the capacity of the cell to repair damaged DNA. However, it is also possible that checkpoints that detect DNA damage and destroy damaged cells also become dysfunctional with age and allow the ovulation of low-quality oocytes. Humans only ovulate ~12 viable oocytes per year, and low-quality or unviable oocytes, which become more common with age, significantly affect fertility.

Our data suggest that indoles not only induce DNA repair in aging or damaged oocytes, thereby facilitating their survival, but also reduce oocyte viability by facilitating cell death, especially in highly damaged or aged cells. The skewing of mammalian oocytes toward cell death and worms toward survival and repair is reflected in differential tuning of indole regulation in these animals. Thus, the regulation by indole appears to be more stringent in mammals perhaps to ensure high-fidelity transmission of genetic information to a limited number of offspring, which are energetically expensive to bear. Thus, in aged mammalian oocytes, indoles promote significant fragmentation and limit blastocyst formation (Fig. 5), possibly to limit transmission of damaged DNA. By contrast, worm oocytes are skewed toward repair and survival to ensure many clonal hermaphroditic offspring (Fig. 2). Although high-fidelity transfer of clonal genetic information is preferred in worms, the tolerances do not appear as stringent as in mammals, as males occur at a frequency of ~0.1%. To ensure genome integrity, indole limits male frequencies and increases embryo viability via repair of damaged DNA. When damage is more severe, for example, with high doses of radiation, most F1 hatchlings are sterile and produce no embryos, and those that do, produce embryos that fail to hatch (Fig. 6). Under these conditions, indole acts as it does in mammals to increase rates of embryo death, in effect increasing the proportion of viable offspring and decreasing the proportion of sterile F1 offspring, which promotes genetic integrity over subsequent generations. Thus, the tuning of the indole rheostat in *C. elegans* and mammals maintains genome integrity, but animal-specific skewing toward death in mammals or repair in worms, except when damage is excessive, serves as a baseline upon which the indole regulation acts.

### The microbiota, indoles, and fecundity in mammals

Our data raise the possibility that the commensal microbiota in the intestine or the vagina regulate oocyte viability both during development within the ovary and following ovulation. In this regard, *Lactobacilli* spp., which produce indole and indole derivatives, have been implicated in vaginal microbiota health by preventing preterm labor (*43*), although a role in oocyte viability has not been studied. Indoles likely play a role at many stages of reproduction. The AhR receptor is expressed throughout both the female and male reproductive tracts, and its removal causes infertility in both sexes (*44*). Activation of indole regulation resulting in DNA repair or fragmentation appears to reduce the probability of fertilizing a damaged oocyte or of allowing a damaged oocyte to develop and may ultimately increase the likelihood of a viable pregnancy while reducing the likelihood of an unviable one. Agents that increase the likelihood of a successful ovulation event are in high demand as part of an IVF protocol. Despite declining oocyte quality in donors over age 30 years, no methodology exists to define whether an oocyte is defective or capable of producing a viable offspring. Compounds such as melatonin and quercetin, which augment DNA repair, have been proposed in this regard (*45*, *46*). Indoles may facilitate IVF by reducing the numbers of unviable oocytes or by promoting repair of viable but damaged oocytes, or both. In addition, indoles may prove useful as biomarkers for the health of the gut or vaginal microbiota and serve as indicators of disorders associated with aneuploidy or reproductive failure.

### The indole/Ahr system as a “capacitor” for genomic diversity

The observations presented here raise the general question of why microbiotas or dietary components acting via indole might regulate aneuploidy. We speculate that a symbiotic relationship between the host and the microbiota may have evolved to regulate youthful homeostasis and ensure replicative fidelity and maintain the microbiota niche. The presence of indole, which also promotes healthspan (*8*), may limit the effects of environmental stressors in worms to maintain genome fidelity, promote hermaphroditic offspring, and preclude sexual reproduction, at least under stable environmental conditions. Conversely, the absence of indole, particularly in the context of environmental stressors or even changes in the microbiota, may promote the formation of aneuploid males in *C. elegans*. Males undergo sexual reproduction with other strains, which amplifies the numbers of males, rapidly expands numbers of progeny, and increases genetic diversity (*47*). Thus, the presence of indoles ensures the high-fidelity transfer of genetic information between generations, whereas its absence enables the population to rapidly adapt within a generation to a stressor. In this sense, indole and its receptor AhR serve as a buffer that maintains genome integrity, especially during homeostatic environmental conditions and as animals age. When the buffering capacity is exceeded by increasing stressor levels or reducing indole levels, for example, during dysbiosis, the system rapidly switches to one that favors outcrossing. The indole/AhR system thus functions analogously to Hsp90 as a means of allowing rapid adaptation to a stressor. Hsp90 has a chaperone activity that buffers silent homeotic mutants in signaling pathways (*48*). Upon heat stress, Hsp90 responds to unfolded proteins, and as the chaperone activity diminishes, signaling pathways are unveiled that induce significant changes in body structure (*48*). Whereas the Hsp90 system has the capacity to unveil preexisting silent homeotic mutations within a population, the indole/AhR system amplifies overall genetic diversity, which can then be fixed once homeostasis and indole levels are reestablished. Notably, the loss of Hsp90 has likewise been associated with aneuploidy induced by heat stress in yeast (*49*) and null mutation in the spineless gene (*ss*), and the AhR ortholog in *Drosophila melanogaster* results in homeotic mutations (*50*). Our data suggest that, similar to Hsp90 (*48*), the indole/AhR system serves as a “capacitor” or “buffer” that permits rapid adaptive responses to stress within a generation via regulation of aneuploidy and ultimately genetic diversity. It is noteworthy that dysregulation of HSP90 signaling has been associated with neoplastic transformation in mammals (*51*). Accordingly, our finding that indoles limit aneuploidy and even promote apoptosis in heavily damaged cells may preclude neoplastic transformation. Indole-3-carbinol, an indole derivative commonly found in cruciferous vegetables, exhibits antitumor activity including triggering apoptosis in tumor cells and reducing tumor invasion and metastasis (*52*). Thus, indoles may link diet and the gut microbiota to neoplastic transformation and even serve as a biomarker whose absence, similar to HSP90, may signal cancer susceptibility.

## MATERIALS AND METHODS

### Bacterial strains

The bacterial strains used in our experiments include *E. coli* MG1655* strain (referred as K12 in this work), which is an efficient colonizer of the murine intestinal tract [obtained from P. Cohen (*53*)], and K12∆*tnaA*, a strain constructed from MG1655* by the deletion of tnaA gene (*tnaA*) (*8*) using the lambda-red-recombinase system (*54*). All bacterial strains were cultured in LB broth (Difco).

### *C. elegans* strains

The following *C. elegans* strains were obtained from the Caenorhabditis Genetics Center: wild-type Bristol strain N2, *ahr-1*(*ju145*), *ahr-1*(*ia3*), *him-5*(*e1490*), *zim-1*(*tm1813*), *zim-1*(*tm1813*), *him-19*(*jf6*), *him-6*(*e1423*), *syp-1*(*me17*), *syp-2*(*ok307*), *htp-3*(*tm3655*), *spo-11*(*me44*), *him-8*(*tm611*), *him-3*(*e1147*), *cep-1*(*gk138*), *hus-1*(*op241*), *cku-70*(*tm1524*), *cku-80*(*ok861*), *mre-11*(*ok179*), *atm-1*(*gk186*), *ced-3*(*n717*), and *ced-4*(*n1162*). The *cep-1*(*gk138*), *him-5*(*e1490*), *cep-1*(*lg12501*), and *him-5*(*e1490*) strains were a gift from B. Derry (*20*).

### Murine strains

C57BL/6J, *ahr* knockout mice (B6;129-Ahrtm1Bra/J), and *p53* knockout mice (B6.129S2-Trp53tm1Tyj/J) were purchased from the Jackson Laboratory. CD1-IGS mice also called Crl:CD1(ICR) were purchased from Charles River. Mice were acclimated for at least 1 week following shipment and before experiments. Animal handling and experimental procedures were in accordance with the *Guide for the Care and Use of Laboratory Animals* and approved by the Emory University Institutional Animal Care and Use Committee.

### X-ray irradiation

Worms, mammalian cells, or mammalian oocytes were x-ray–irradiated at the doses indicated in the text using an RS-2000 RadSource Technologies device at ~1.2 Gy/min.

### *C. elegans* growth conditions

All *C. elegans* strains were maintained on Nematode Growth Medium (NGM) at 16°C (*55*, *56*) with OP50 as food source. Gravid *C. elegans* adult worms were bleached on the assay plates as described in the Wormbook to obtain larvae for the experiments. For most assays, worms were grown under either vehicle or indole conditions.

### Indole administration in *C. elegans*

Synthetic indole (Sigma-Aldrich) dissolved in methanol (Sigma-Aldrich) was added to NGM medium after sterilization and cooling to ~55°C to a final concentration of 100 μM. The K12 or K12∆*tnaA* bacterial strains that were grown overnight in LB broth at 37°C to an OD_600_ (optical density at 600 nm) of 0.8 to 1.0 were used to seed the assay plates and served as food for *C. elegans*. Seeded plates were kept at room temperature for at least 12 hours before use in assays. Previous work showed that worms exhibit maximum healthspan when they are grown in the K12 supplemented with 100 μM indole in the agar, compared to those grown in either K12 or K12∆*tnaA* + indole (*8*). Thus, to obtain maximal indole effect, we used this concentration in most worm experiments in this paper, unless otherwise noted. As metabolism of tryptophan by K12 generates indole together with other indole species (*13*), we refer to the active agent as “indoles” throughout this work. The concentration of exogenous indole used here corresponds to physiological levels found in the mammalian intestinal tract; indoles are present in millimolar concentrations in mammalian intestine (*8*) and in agar on which indole-secreting bacteria are plated (*13*). As a control, methanol (vehicle) was added to NGM together with the K12∆*tnaA* bacterial strain, which does not produce indole. In all experiments, worms were grown without 5-flurodeoxyuridine, a chemical routinely used to inhibit appearance of the progeny from next generation.

### *C. elegans* heat and radiation stress

*C. elegans* were grown from embryos (egg) to desired developmental stage in vehicle or indole conditions as described above. For heat stress, worms were subjected to 30°C heat stress for 6 hours. Stressed worms were then transferred to 16°C for recovery, and their progeny (brood) was monitored for incidence of males and dead embryos. For radiation stress, worms were subjected to different doses of X-irradiation in an RS-2000 RadSource Technologies device at ~1.2 Gy/min. Irradiated worms were then transferred to 16°C, and their progeny was monitored for males and dead embryos.

### *C. elegans* aging experiments

L4 worms grown as described in the text were transferred every other day until the end of the reproductive cycle, which occurred on about the fifth day of adulthood (D5). Progeny laid until D3 of adulthood was considered as F1 from young worms, and those from D3 to D5 were considered as F1 from old worms. Dead embryos and male numbers were determined in both groups.

### Embryonic lethality and male frequency calculations

*C. elegans* generally reproduce as hermaphrodites, generating sperm during larval development and using those sperm to fertilize oocytes generated during adulthood, a process resulting in low embryo lethality and clonal hermaphroditic offspring. In oocytes, aging and exposure to stressors, including heat and X-irradiation, increase the rate of nondisjunction on the X chromosome, which, upon fertilization, results in increased numbers of aneuploid progeny, which are male and reproduce sexually by mating with hermaphrodites of the same or different genetic backgrounds (*57*, *58*). Notably, loss-of-function alleles in many meiotic genes result in a high incidence of males among the progeny, called the “*him*” phenotype (*11*, *59*). Analysis of these mutants led to identification of genes governing critical stages of chromosomal segregation and DNA repair during meiosis (*19*–*22*). Aging, stressors, and many *him* mutants also induce aneuploidy on autosomes, which results in dead embryos. Males are distinguished from the hermaphrodites by their distinct body shape and tail structure (*11*). Male percentage in a brood was calculated as (number of males/total live progeny counted) × 100. An egg/embryo was judged as dead if it failed to hatch after more than 24 hours. Dead embryo frequency was then calculated as (number of dead embryos/total embryos laid) × 100, where total embryos equaled live progeny plus dead embryos. Because males and dead embryos without stressors were relatively rare events, their values were enumerated and presented as combined value from all replicates of all independent experiments showing similar trends. Data involving stressor-induced dead embryos in *C. elegans* where the frequency is higher are presented as values from individual replicates of the experiment, and 95% confidence intervals presented show the extent of inter-replicate variability when data from independent experiments were combined.

### Transgenerational embryo viability assay

*C. elegans* were grown from embryos (egg) to D1 of adulthood under vehicle (control) or indole conditions as described above. These F0 worms were subjected to X-irradiation at different doses (60 or 120 Gy). The survival of their embryos (F1s) was then scored. The F1s from the vehicle control condition were allowed to grow in vehicle and indole F1s under indole conditions across generations. No stress was introduced to F1s or subsequent generations, and indole and vehicle treatment was maintained across the generations. The viability of F2 embryos from these F1s was enumerated. Most of the F2s appeared sick, displayed developmental arrest, or were sterile. The nonsterile F2s were further evaluated, and the viability of their embryos (F3) was scored. As in other embryo viability assays, viability was scored as the brood size of each worm, and the data shown represent the combined values of all replicates from all independent experiments.

### Acridine orange and cell cycle arrest assays

Worms were subjected to acridine orange staining to measure cell corpses as previously described (*60*). For arrest experiments, worm gonads were dissected and stained with DAPI to visualize and quantify mitotic cells as previously described (*60*).

### Mammalian cell culture and maintenance

NIH/3T3 cells were maintained in Dulbecco’s modified Eagle’s medium (DMEM) supplemented with 10% fetal bovine serum (FBS) as previously described (*61*).

### Cytochalasin B micronucleus assay

Mouse 3T3 expressing nuclear H2B-GFP was used to allow easy visualization of nuclei by fluorescence microscopy. Cells were plated on coverslips and treated with 100 μM ICA or vehicle [dimethyl sulfoxide (DMSO)] for 24 hours, followed by x-ray irradiation at the doses noted. Cytochalasin B (1.5 μg/ml) was added immediately after irradiation, and cells were washed with phosphate-buffered saline (PBS) and fixed 48 hours later, and coverslips were mounted on slides for visualization. The percentage of binucleated cells with one or more micronuclei was determined.

### Immunofluorescence (γ-H2AX staining)

3T3 cells were plated on coverslips and treated with 100 μM ICA or vehicle (DMSO) for 24 hours, followed by x-ray irradiation at the doses noted. Cells were fixed in 2% formaldehyde at various time points after irradiation and permeabilized in Triton X-100 for immunofluorescence analysis as previously described (*62*). Nuclei were visualized with DAPI (1 g/ml; Sigma-Aldrich, St. Louis, MO), and DSBs were visualized with anti–phospho-histone H2AX (Ser^139^), clone JBW301 (Sigma-Aldrich).

### Experimental protocols with mice

#### ICA administration

ICA (Sigma-Aldrich) was delivered daily by oral gavage at a dose of 150 mg·kg^−1^·per day in a vehicle of DMSO/PEG400 (polyethylene glycol, molecular weight 400)/5% citric acid (1:4.5:4.5), as previously reported (*18*).

#### K12 colonization

Mice were colonized with *E. coli* K12 or K12ΔtnaA strains, and colonization was verified as previously reported (*8*).

#### Splenocyte isolation

Splenocytes were isolated from different mouse strains as previously reported (*63*). Briefly, cells were obtained by macerating spleen through a cell strainer in a petri dish of DMEM/10% FBS/Pen/strep medium.

#### Oocyte collection

Females at various ages were induced to superovulate by injections of 7.5 IU PMSG and 7.5 IU hCG administered 48 hours apart and euthanized by cervical dislocation 13 hours after hCG injection. Oviducts were removed quickly and placed into a preequilibrated K-RVFE-50 medium (COOK Medical). Oocytes were collected in M2 medium (Sigma-Aldrich, USA) as described before (*27*). For in vivo postovulatory aging experiments, mice were euthanized 24 hours after hCG, and oocytes were collected in M2 medium.

#### In vitro fertilization

Intact cumulus oocyte complexes were released from the excised oviducts into CARD medium (Cosmo Bio USA) 13 hours after hCG. Fresh sperm were obtained from the cauda epididymides of 3- to 4-month-old males and were suspended in a dish containing a drop of mouse sperm preincubation medium (FERTIUP, Cosmo Bio USA) covered with silicon oil. They were then incubated for 30 min at 37°C with 5% CO_2_ in the air. After preincubation, the sperm suspensions were added to a drop of CARD medium containing oocytes and incubated at 37°C with 5% CO_2_. After 4 to 6 hours, the inseminated oocytes were washed three times and cultured in drops of K-RVFE-50 medium (COOK Medical) with calcium. Eight to 10 hours after insemination, the formation of pronuclei was observed.

### Comet assays with 3T3 cells, splenocytes, and oocytes

Alkaline comet assays were performed using the R&D Systems CometAssay single-cell gel electrophoresis assay as described by the manufacturer. Briefly, individual cells are embedded in a thin agarose gel on a microscope slide. All cellular proteins are then extracted from the cells by lysing. The DNA is allowed to unwind under alkaline/neutral conditions. Following the unwinding, the cell/DNA is subjected to electrophoresis, allowing the broken DNA fragments or damaged DNA to migrate away from the nucleus. After staining with a DNA-specific fluorescent dye such as ethidium bromide or propidium iodide, the “cell/DNA” is imaged, and the amount of fluorescence in head and tail and length of tail was determined. The image obtained resembles a “comet” with a distinct head and tail. The head is composed of intact DNA, while the tail consists of damaged (single-strand breaks or DSBs) or broken pieces of DNA. The extent of DNA liberated from the head into the tail of the comet is directly proportional to the amount of DNA damage.

Various cell types were evaluated in comet assays. 3T3 cells were treated with ICA or vehicle for 24 hours, irradiated at the doses indicated, and trypsinized at either 5 or 20 min. After irradiation, cells were washed once in ice-cold 1× PBS (Ca^2+^ and Mg^++^ free) and suspended in ice-cold 1× PBS (Ca^++^ and Mg^++^ free) at 1 × 10^5^ cells/ml. Cells were mounted on comet slides, electrophoresed, and stained with Sybr Gold, according to manufacturer specifications. Slides were imaged by epifluorescence microscopy, and images were collected of all detectable comets on each slide. For splenocytes and oocytes, isolation was performed as described as above, X-irradiated in RPMI and M2 media, respectively, and the comet assay was performed as above. In experiments where mice were treated with vehicle or ICA, the medium did not contain ICA or vehicle after cells were isolated from the animal, during radiation ex vivo, or after radiation. However, for experiments with untreated animals, cells were treated with ICA/vehicle ex vivo, and the medium contained ICA or vehicle in all postisolation manipulations. For 3T3 cells and splenocytes, images were analyzed using Open Comet software (https://cometbio.org/) to determine percent tail DNA, olive moment, and tail moment. Because of the difference in cell size, oocytes were not easily recognized by Open Comet; thus, manual measurements were obtained using Intelligent Imaging Innovations Slidebook software (V6.1). For each oocyte comet, masks were used to calculate the area and intensity of the head of the comet alone, as well as that of the whole comet (head + tail). Percent tail DNA was calculated as follows: (i) head area × head intensity = head DNA; (ii) total comet area × total intensity = total comet DNA; (iii) total comet DNA − head DNA = tail DNA; and (iv) (tail DNA/total comet DNA) × 100 = % tail DNA.

### Statistical analysis

In *C. elegans*, males and dead embryos without stressors are rare events. Thus, their values were enumerated and presented as the combined value from all replicates of all independent experiments showing similar trends. The *P* values in this situation were obtained with a chi-square test. Data involving stressor-induced dead embryos in *C. elegans* (where the frequency is relatively higher) are presented as values from individual replicates of the experiment, and 95% confidence intervals presented show the extent of inter-replicate variability when data from independent experiments were combined. The *P* values in this situation were obtained with a Mann-Whitney test. Other data were analyzed by a Mann-Whitney test or Kruskal-Wallis analysis of variance (ANOVA), which yielded an exact *P* value. A value of less than 0.05 was considered significant. The method of analysis is described in the figure legends.

## Notes

View/request a protocol for this paper from *Bio-protocol*.
