# Supplementary material for: A microbiota and dietary metabolite integrates DNA repair and cell death to regulate embryo viability and aneuploidy during aging
Source: Sci Adv. 2023 Feb 24;9(8):eade8653. doi: 10.1126/sciadv.ade8653 (PMC9956122; doi:10.1126/sciadv.ade8653)
Supplement: Supplementary file 1 — Figs. S1 to S4 [file sciadv.ade8653_sm.pdf]

Science Advances
AAAS

Supplementary Materials for

A microbiota and dietary metabolite integrates DNA repair and cell death to regulate embryo viability and aneuploidy during aging

Robert Sonowal *et al.*

Corresponding author: Daniel Kalman, dkalman@emory.edu

Sci. Adv. **9**, eade8653 (2023)
DOI: 10.1126/sciadv.ade8653

This PDF file includes:

Figs. S1 to S4

Supplementary Figures

A

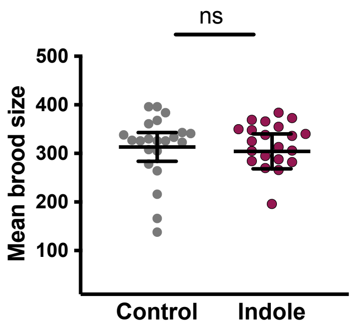

B

Strains	Average Total eggs/Brood ± SD (# of broods scored)		*P* value
	Control	Indole	
him-5 (e1490)	251.5 ± 67.5 (36)	233.9 ± 62.9(36)	0.68
zim-1(tm1813)	243.2 ± 33.2(10)	213 ± 36.8(10)	0.12
zim-2(tm574)	191.5 ± 77.4(14)	227.3 ± 60(14)	0.25
him-19(jf6)	NA	NA	NA
him-6(e1423)	NA	NA	NA
syp-1(me17)	237.3 ± 53.4(36)	165.3 ± 68(36)	<0.00001
syp-2(ok307)	234.3 ± 49(12)	183.1 ± 30.3(12)	0.02
htp-3(tm3655)	238.7 ± 61.7(25)	200.7 ± 44.7(25)	0.02
spo-11(me44)	280.6 ± 51.4(16)	251.6 ± 56.2(16)	0.1
him-8(tm611)	257.2 ± 64.6(10)	265.2 ± 42.1(10)	0.32
him-3(e1147)	251.8 ± 86.3(20)	256.2 ± 49.7(20)	0.98

Figure S1. Effects of indoles derived from commensal *E. coli* on brood size of N2 and meiotic mutants. A & B, Mean brood size of N2 or meiotic mutant worms grown either in Control or Indole. A brood is defined as the total number of embryos (both live and dead) laid by one adult worm. All values are obtained by combining results from at least two independent experiments showing similar trend. Values presented here are mean values ± SD and the *P*-values were calculated with Mann-Whitney test. **P*<0.05, ***P*<0.01, ****P*<0.001, *****P*<0.0001.

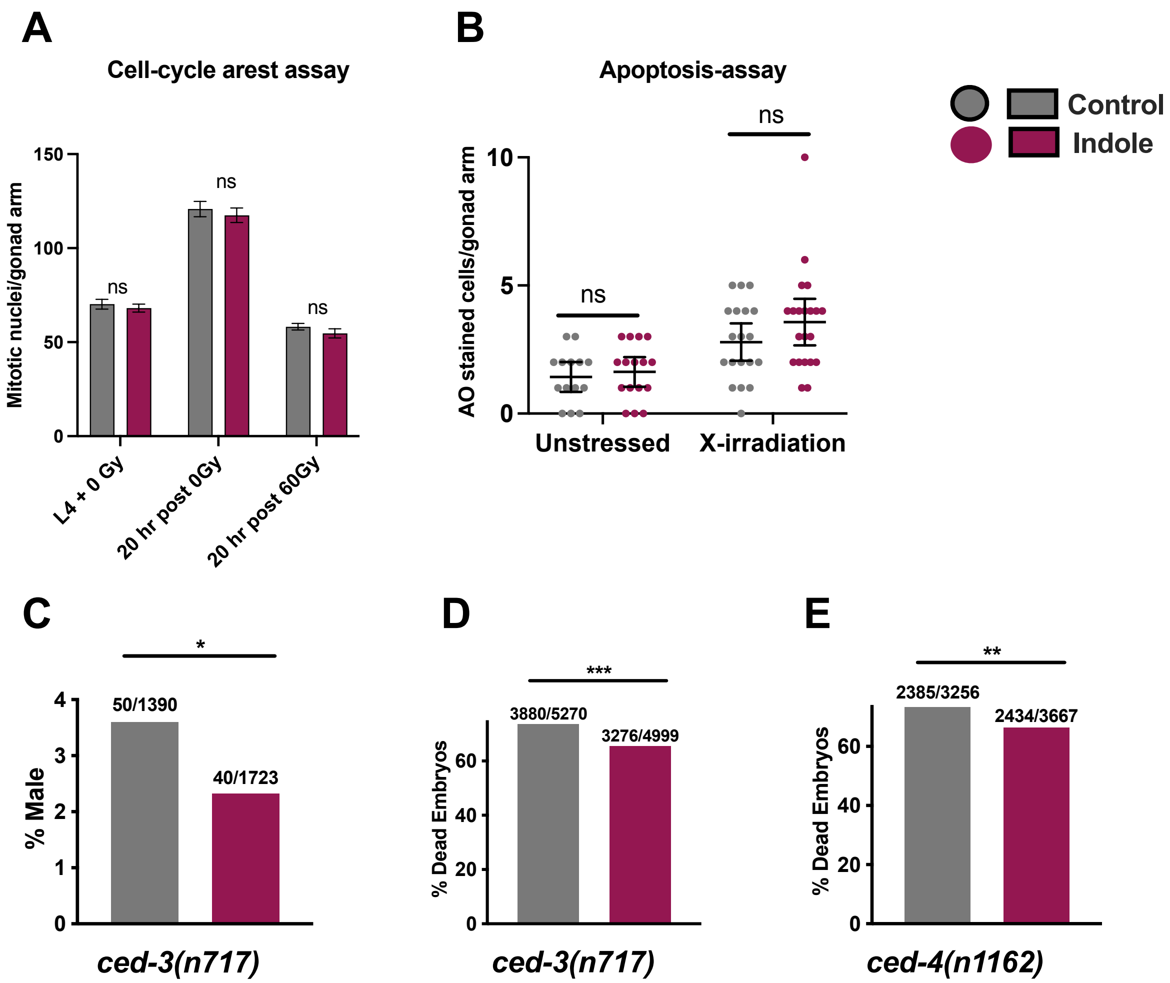

Figure S2. Indoles don't influence cell cycle arrest and apoptosis post X-radiation in *C. elegans*. **A**, Numbers of DAPI stained nuclei in the distal tip region of the gonad of N2 worms grown till L4 larval stage in either Control or Indole, subjected to either 60Gy or no X-ray, scored 20hrs post radiation or without radiation. **B**, Acridine orange-stained nuclei scored in pachytene region of gonads of N2 worms grown till embryo to L4 stage in Control or indole condition, subjected to 60 Gy or no stress and scored 20 hrs post radiation. **C-E,** Frequencies of males (**C**) and dead embryos (**D & E**) produced by *ced-3(n717)* (**C & D**) and *ced-4(n1162)* (**E**) worms grown either in Control or Indole and exposed to 60 Gy X-ray. Values on the bars in **C** represents "male numbers/total adults counted" and "numbers of dead embryos/total embryos" in **D, E**. All values are obtained by combining results from at least two independent experiments showing similar trend. Values presented in **A & B** are mean values ± 95% CI and the *P*-values were calculated with Mann-Whitney test. *P*-values in **C-E** were calculated with Chi-squared test. *$P<0.05$, **$P<0.01$, ***$P<0.001$, ****$P<0.0001$.

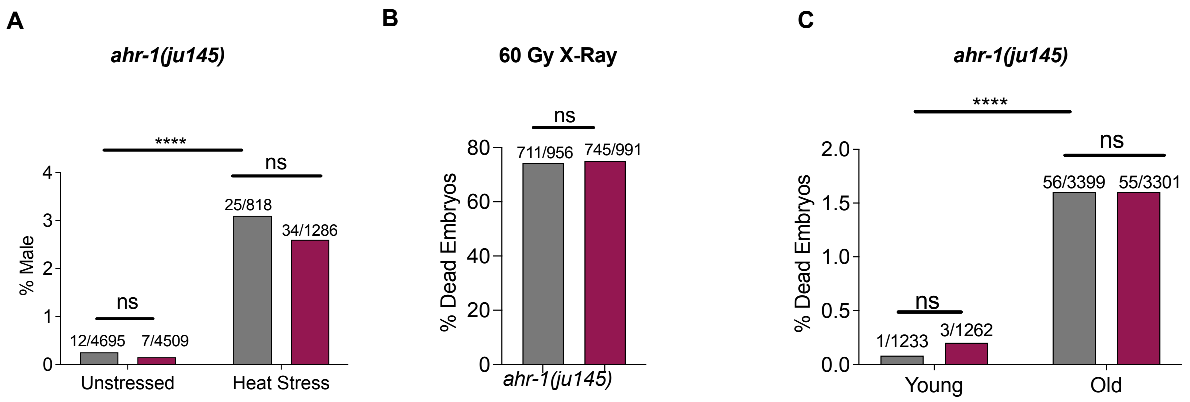

Figure S3. Indoles require Ahr-1 to limit embryonic lethality and aneuploidy in *C. elegans*. **A**, Frequency of males in the progeny from *ahr-1(ju145)* worms grown either in control or indole, subjected to either heat or no stress. **B**, Frequency of dead embryos from *ahr-1(ju145)* worms grown either in control or indole, subjected to 60 Gy X-ray. **C**, Frequency of dead embryos in the progeny of Young (D1-D3 of adulthood) and Old (D3-D5 of adulthood) *ahr-1(ju145)* worms grown either in Control or Indole conditions. Values on the bars in **A** represent "number of males/total adults counted". Values on the bars in **B & C** represent "number of dead embryos/total embryos counted". All values are obtained by combining results from at least two independent experiments showing similar trend. *P*-values were calculated with Chi-squared test. *$P<0.05$, **$P<0.01$, ***$P<0.001$, ****$P<0.0001$.

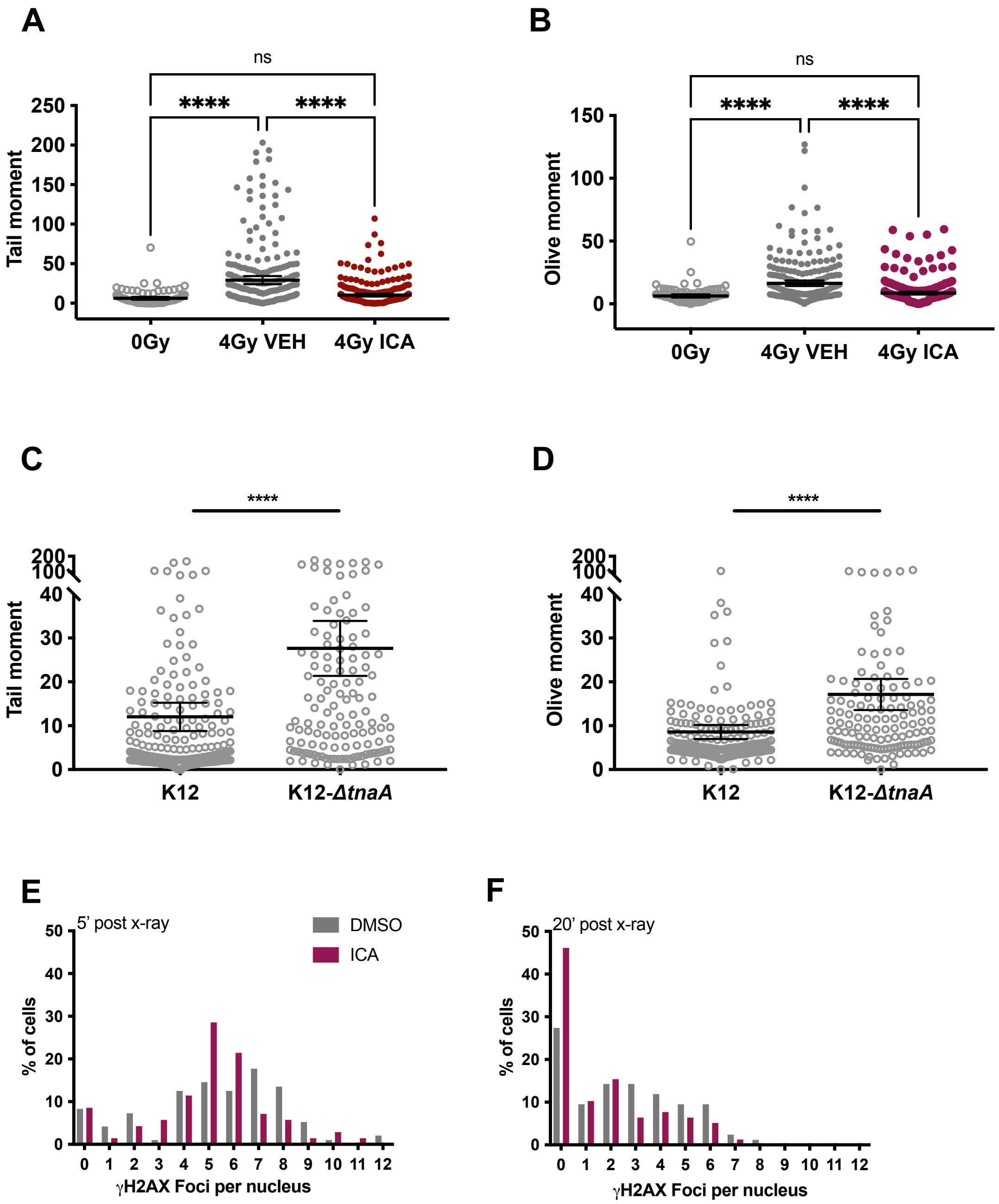

Figure S4. A & B, Tail moment and olive moment in the comets of 3T3 cells, treated with vehicle or ICA for 24 hrs and subjected to 0Gy, 4Gy of X-irradiation (n>102 comets/condition). **C & D**, Tail moment and olive moment in the comets of splenocytes isolated from 3 months old C57BL/6 mice colonized with either K12 or K12ΔtnaA for 60 days. Splenocytes were X-irradiated at 4Gy, and comet assay performed 20' post radiation (n>200 comets/condition). Values presented are mean values ± 95% CI and the *P*-values were calculated with Mann-Whitney test. *$P<0.05$, **$P<0.01$, ***$P<0.001$, ****$P<0.0001$.
